# A Data-Driven Learning Method for Constitutive Modeling: Application to Vascular Hyperelastic Soft Tissues

**DOI:** 10.3390/ma13102319

**Published:** 2020-05-18

**Authors:** David González, Alberto García-González, Francisco Chinesta, Elías Cueto

**Affiliations:** 1 Aragon Institute of Engineering Research, Universidad de Zaragoza, 50018 Zaragoza, Spain; gonzal@unizar.es; 2 Laboratori de Càlcul Numèric, E.T.S. de Ingeniería de Caminos, Universitat Politècnica de Catalunya, 08034 Barcelona, Spain; berto.garcia@upc.edu; 3 ESI Group Chair, Processes and Engineering in Mechanics and Materials (PIMM) Laboratory, Arts et Metiers Institute of Technology, 75013 Paris, France; Francisco.Chinesta@ensam.eu

**Keywords:** machine learning, manifold learning, topological data analysis, GENERIC, soft living tissues, hyperelasticity, computational modeling

## Abstract

We address the problem of machine learning of constitutive laws when large experimental deviations are present. This is particularly important in soft living tissue modeling, for instance, where large patient-dependent data is found. We focus on two aspects that complicate the problem, namely, the presence of an important dispersion in the experimental results and the need for a rigorous compliance to thermodynamic settings. To address these difficulties, we propose to use, respectively, Topological Data Analysis techniques and a regression over the so-called General Equation for the Nonequilibrium Reversible-Irreversible Coupling (GENERIC) formalism (M. Grmela and H. Ch. Oettinger, Dynamics and thermodynamics of complex fluids. I. Development of a general formalism. Phys. Rev. E 56, 6620, 1997). This allows us, on one hand, to unveil the true “shape” of the data and, on the other, to guarantee the fulfillment of basic principles such as the conservation of energy and the production of entropy as a consequence of viscous dissipation. Examples are provided over pseudo-experimental and experimental data that demonstrate the feasibility of the proposed approach.

## 1. Introduction

Computational (bio-)mechanics is not absent of the data “fever”. Even if the so-called data-driven computational mechanics discipline presents distinctive features over what is commonly known as “big data”, the possibility of employing raw experimental data to perform simulations has attracted the attention of many researchers recently [1,2,3,4,5,6,7,8,9]. In these approaches, basic equations—those with a higher epistemic value, such as equilibrium or compatibility—are kept, while those which are frequently phenomenological in nature—constitutive equations—are substituted by experimental data, and hence, the name of this family of methods. In a related approach, the so-called equation-free approach substitutes the constitutive law of a material not by experimental data, but by a pseudo-experimental result coming from microscale, first-principle computations [10].

This approach allows us to avoid complex, costly—and often unsuccessful—parameter fitting to obtain the precise form of a constitutive equation. On the other hand, widely accepted constitutive equations have been built under rigorous standards that give rise to the automatic satisfaction of first principles. This is not evident for data-driven approaches.

The presence of noise, or large variance among experiments in the data, somehow complicates the task of obtaining results that do not violate the laws of thermodynamics. Previous works in the field address the presence of noise in the results, but do not guarantee the satisfaction of basic thermodynamic principles [11,12]. This effect is particularly important in the field of bioengineering, where soft living tissues present a very important dispersion of the obtained parameter values from sample to sample, see for instance [13,14] and references therein.

Another important issue is that of the highly nonlinear anisotropic behavior of soft living tissues, which are able to show besides, inelastic and viscoelastic responses. Many works have been published in the last decades. In this regard, these behaviors have been traditionally modeled and characterized as (possibly visco-)hyperelastic anisotropic materials by means of strain energy density functions, see for example, in [15,16,17,18,19,20] and references herein. Inelasticity in the data-driven setting has been analyzed in a number of previous works [21,22,23]. Even the more daring approaches employ deep learning to relate medical images to mechanical properties of the tissue [24]. In none of the mentioned works is a study made by which the thermodynamic consistency of the obtained results is guaranteed. If we combine this possibility with the presence of noise in the data, the chance of violating first principles comes into play.

Different approaches exist to guarantee a rigorous thermodynamic compliance. For instance, Raissi et al. developed the so-called *physics-informed deep learning* method for the solution of partial differential equations [25]. A similar approach has also been developed to model turbulence [26] or, in general, the generalized Langevin equation [27]. The authors have recently developed an alternative approach based on the so-called General Equation for the Nonequilibrium Reversible-Irreversible Coupling (GENERIC) [28,29,30]. The GENERIC equation, which will be described next, constitutes a generalization of the Hamiltonian description of physical systems under nonequilibrium settings. The employ of the GENERIC formalism thus guarantees the correct fulfillment of the first and second principles of thermodynamics, and gives rise to a machine learning method valid for the description of the system at any level, from the molecular dynamics governed by Newtonian laws to the invariant-based description at the thermodynamics level [31,32].

The proposed method can be seen as a particular instance of the continuous dynamical system approach to machine learning, first proposed by W. E and coworkers [33,34]. In the quest for a theoretical framework for deep learning, a parallelism has been established between deep neural networks (DNNs) and continuous dynamical systems. DNNs are thus viewed under this prism as a discretization of a continuous dynamical system that maps the set of observations to a nonlinear function that fits the data [33]. This alternative view opens the door to the enforcement of desired properties to the learning processes; for instance, to ask the resulting map to posses a Hamiltonian structure, among others. In this work, we force the resulting map, which is found by regression in a piecewise polynomial manner, to obey a GENERIC description. This will ensure, as mentioned earlier, the fulfillment of the first and second principles of thermodynamics. To show its strong potentiality in the field of mechanical modeling of biological tissues, the method thus developed will be applied as a proof of concept to the characterization of the passive mechanical behavior of porcine carotid tissue. This behavior turns out to be—under the experimental setting considered here—elastic, highly nonlinear, anisotropic at finite strains, and often modeled under the framework of hyperelasticity.

The outline of the paper is as follows: Section 2 details the proposed data-driven model and the developed tests for validation. In Section 2.1, we describe the proposed machine learning technique to fit the mechanical responses of biological materials. To fit the well-known hyperelastic response in soft living tissues, the thermodynamically consistent GENERIC approach (describing the physics of the problem) and the subsequent machine learning procedure (constitutive manifold) is detailed here. Next, since numerical fitting of such tissues is strongly subjected to dispersion and averaging (mainly provoked by experimental procedures, environmental issues during the manufacturing, and testing process), a treatment of the noise and dispersion by means of Topological Data Analysis (TDA) is proposed in Section 2.2. Our presented approach is tested upon both a pseudo-experimental data set and experimental tests, described in Section 2.3 and Section 2.4, respectively. The numerical fitting results of both experiments are shown in Section 3. The paper ends in Section 4 with a discussion of the obtained results on the use of the proposed GENERIC-TDA methodology on the mechanical modeling of biological tissues.

## 2. Material and Methods

### 2.1. A GENERIC Approach to the Learning Procedure

Recently, W. E and coworkers established a very useful parallelism between DNNs and dynamical systems [33]. Consider a system governed by some state variables $z(z_{0},t):I\to S$, $z\in C^{1}\left(0,T\right]$, such that its time evolution is established in the form

(1) $$\frac{dz}{dt}=f\left(z,t\right),\;\;z\left(0\right)=z_{0}$$

where *f* is, in general, a nonlinear function—otherwise, the method is of little interest. S represents the phase space of the system (a set of judiciously chosen variables that both describe the energy of the system and can be measured experimentally). I=(0,T] represents the considered time interval.

For the selected time horizon *T*, the flow map

$$z_{0}\to z\left(z_{0},T\right)$$

is a nonlinear function of z. The dynamical system approach to supervised learning consists in determining *f* so that the resulting flow map is able to reproduce the experimental data. This parallelism offers the advantage of the vast knowledge developed so far in the field of dynamical systems, which could help us in developing both theoretical insights about DNNs and also to devise alternative routes for developing efficient learning strategies. DNNs can be thought of as discretized dynamical systems, such that every time step corresponds to a layer of the DNN.

In this work, we consider viscous-hyperelastic materials. Therefore, the learned function *f* must satisfy certain well-known principles dictated by thermodynamics. In the hyperelastic case, the right choice for *f* could arise from Hamiltonian mechanics, i.e.,

$$\frac{dz}{dt}=L\left(z\right)\nabla E\left(z\right)$$

where *E* represents the Hamiltonian or, in other words, the energy of the system. L(z) represents the so-called Poisson matrix, a skew-symmetric matrix that depends on z, in general, but that results to be constant in many cases.

Should the system of interest not be conservative (or Hamiltonian), a new potential needs to be introduced in the formulation—entropy—giving rise to the GENERIC formalism [30]

(2) $${\dot{z}}_{t}=L\left(z_{t}\right)\nabla E\left(z_{t}\right)+M\left(z_{t}\right)\nabla S\left(z_{t}\right),\;\;z\left(0\right)=z_{0}$$

For Equation (2) to represent valid nonequilibrium thermodynamic processes, it must be supplemented with the so-called degeneracy conditions, i.e.,

(3a) $$\begin{array}{cc} L(z)\cdot \nabla S(z) & =0 \end{array}$$

(3b) $$\begin{array}{cc} M(z)\cdot \nabla E(z) & =0 \end{array}$$

If, as stated before, L is skew-symmetric; and choosing M to be symmetric, positive semidefinite, we obtain

$$\dot{E}\left(z\right)=\nabla E\left(z\right)\cdot \dot{z}=\nabla E\left(z\right)\cdot L\left(z\right)\nabla E\left(z\right)+\nabla E\left(z\right)\cdot M\left(z\right)\nabla S\left(z\right)=0$$

i.e., one ensures the conservation of energy in closed systems.

In turn,

$$\dot{S}\left(z\right)=\nabla s\left(z\right)\cdot \dot{z}=\nabla S\left(z\right)\cdot L\left(z\right)\nabla E\left(z\right)+\nabla S\left(z\right)\cdot M\left(z\right)\nabla S\left(z\right)\geq 0$$

which is equivalent to the fulfillment of the second principle of thermodynamics. Thus, we notice how, by leveraging the dynamical systems equivalence, we efficiently enforce the conservation of energy and the production of entropy. For an in-depth discussion of the implications of the choice of phase space variables z, we refer the interested reader to our previous works in the field, [31,32]. In general, it is well-known that neither a particular choice of variables is needed, nor a particular scale for the description of the system at hand. The only need is that the variables in the phase space would be able to account for the energy of the system. That is why, in general, a Langevin equation is not suitable for this purpose, see [35] and references therein.

Following the dynamical systems equivalence, the next step consists in the determination, by regression from data, of the form of the elements of the GENERIC description of the dynamics, i.e., L, M, ∇E(z), and ∇S(z). To do so, assume a standard finite difference discretization of the time derivative,

(4) $$\frac{z_{n+1}-z_{n}}{\Delta t}=L\left(z_{n+1}\right)\mathrm{DE}\left(z_{n+1}\right)+M\left(z_{n+1}\right)\mathrm{DS}\left(z_{n+1}\right)$$

where we denote $z_{n+1}=z_{t+\Delta t}$ and where L and M are the discrete version of the Poisson and friction operators, respectively. DE and DS represent the discrete gradients. In general, matrix L is constant over the process, while matrix M frequently varies.

While there exist machine learning techniques that are able to provide with the precise expressions of the terms involved in a particular PDE from data [2], we pursue a purely numerical route, in which we assume that the elements of the GENERIC formula have a particular manifold structure which is to be unveiled by our method. This is the concept of constitutive manifold that we first stated in our previous works [4,7,36].

The dynamical systems approach to the problem will thus consist of solving the following (possibly constrained) minimization problem within a time interval J⊆I:

(5) $$\mu^{*}=\left\{L,M,\mathrm{DE},\mathrm{DS}\right\}=\underset{\mu }{arg min}||z\left(\mu \right)-z^{\mathrm{meas}}\left|\right|$$

with $z^{\mathrm{meas}}\subseteq Z$, a subset of the total available experimental results. L,M,DE,DS will in general be dependent on z, and therefore the choice of size of $z^{\mathrm{meas}}$ (in other words, the number of regressions performed along the time interval, or the number of layers in the DNNs equivalent) will affect the accuracy of the method. An analysis of the influence of this choice was made in [31].

We finally approximate z by employing piecewise polynomials, so that gradient operators can be cast in matrix form, à la finite elements, as

(6a) $$\begin{array}{cc} \mathrm{DE} & =Az \end{array}$$

(6b) $$\begin{array}{cc} \mathrm{DS} & =Bz \end{array}$$

where A and B represent the discrete, matrix form of the gradient operators leading to DE and DS, respectively.

The GENERIC description of a hyperelastic material is well known [37]. Indeed, for the Hamiltonian, conservative part of the constitutive equation, we have

$$z\left(x,t\right)={\left[x\left(X,t\right),p\left(X,t\right)\right]}^{⊤}$$

where $x=\phi \left(X\right)\in \Omega_{t}\subset R^{3}$ represents the deformed configuration of the solid, $\Omega_{t}$ represents the deformed configuration of the solid at time *t*, and $p\in R^{3}$ represents the material momentum density. In this case,

$$\dot{z}=\left[\begin{array}{c} \dot{x}\\ \dot{p} \end{array}\right]=L\nabla E=\left[\begin{array}{cc} 0_{3\times 3} & I_{3\times 3}\\ -I_{3\times 3} & 0_{3\times 3} \end{array}\right]\left[\begin{array}{c} \frac{\partial E}{\partial x}\\ \frac{\partial E}{\partial p} \end{array}\right]$$

The total energy of a hyperelastic body is known to be the sum

E=W+K

of elastic and kinetic energies. Here, we assume a strain energy density potential *w* of the form

$$W=\int_{\Omega_{0}}w\left(C\right)\;d\Omega$$

where $\Omega_{0}$ represents the undeformed configuration of the solid and C represents the right Cauchy-Green deformation tensor. In a general isotropic case, the strain energy density would take the form w=w(X,C,S). If the material under consideration is isotropic hyperelastic, we simply write w=w(C). In turn, the kinetic energy will be

$$K=\int_{\Omega_{0}}\frac{1}{2\rho_{0}}{\left|p\right|}^{2}\;d\Omega$$

Therefore, by numerically identifying the form of the energy potential, one readily observes that the conservative part of the usual constitutive hyperelastic law is found. If, in addition, the material shows a viscous dissipative behavior, the precise form of the entropy potential is to be found.

Once the learning method has been developed, we describe next how the experimental campaign was accomplished.

### 2.2. Treatment of Dispersion and Noise in Data

One of the most important aspects to consider when dealing with soft living tissues is the importance of the dispersion of experimental results. This will be highlighted in Section 3 below. The main objective of the proposed technique is the determination of a constitutive manifold for the term of the GENERIC description of the physical phenomena, this type of dispersion needs to be treated efficiently.

**Figure 1:**
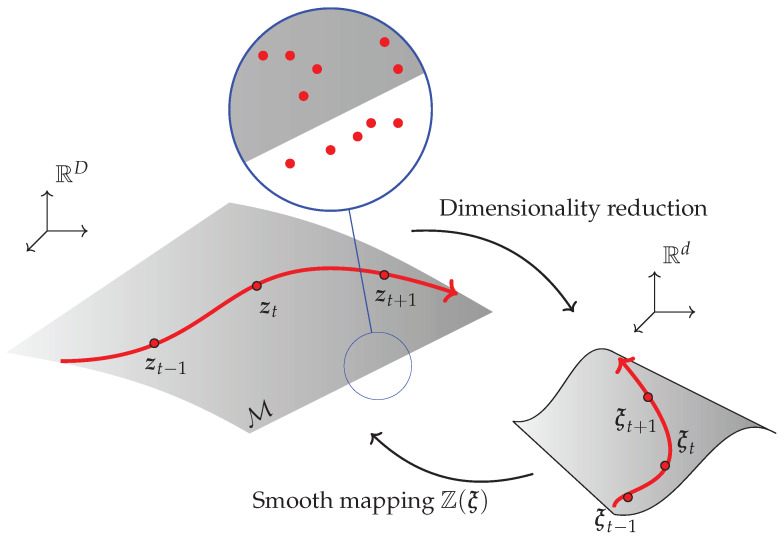
Hypothesis about the existence of a constitutive manifold in which the experimental results live. Despite the noise in the data, Topological Data Analysis (TDA) techniques will help us in unveiling the true geometry of the manifold in the high-dimensional setting, which will later be embedded onto a low dimensional space for the ease of computations.

To this end, we suggest to employ Topological Data Analysis (TDA) [38,39]. Behind the proposed method is the assumption of the existence of a constitutive manifold, a concept that we first introduced in [4]. Our set of experimental measurements, in the most general case, is assumed to be composed by *D*-tuples (D=9 for this particular case) of the type

$$S=\{z=\left(U,P\right)\in \left(R^{3}\times R^{6}\right)\}$$

where U represents the (right) stretch tensor and P the first Piola–Kirchhoff stress tensor. These experimental values are assumed to form a constitutive manifold in a high-dimensional space, see Figure 1. These values are, however, noisy. TDA is nevertheless able to extract the underlying geometry of this manifold, which will later be embedded onto a low-, *d*-dimensional manifold for interpolation purposes.

TDA seeks to find the underlying topological structure of data. To this end, it employs a distance parameter *R* between experimental points. As we make *R* grow, points at distances lower than *R* will be connected by edges, triangles, and tetrahedra—in general, by *k*-simplexes, respectively, in one, two, three, …, *k* dimensions. As simplexes appear, they form simplicial complexes. The study of the formation of this simplicial complex structure in the data is precisely the objective of TDA. The optimal *R* parameter that best describes the topology of the data is found by resorting to the so-called persistence diagrams.

**Figure 2:**
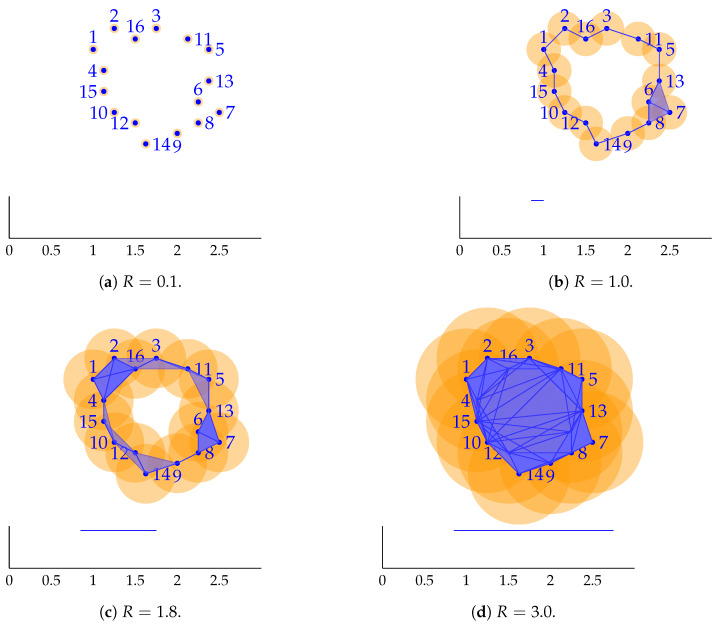
Interpretation of TDA. Orange circles have diameter *R*, the topology parameter. Below each simplicial complex, the bar code corresponding to dimension 1 holes) is represented. (**a**) For a sufficiently small *R* parameter, say, 0.1, only a collection of data points is visible, with no topology at dimension 1. (**b**) Increasing *R* to 1.0 makes the first circular topology appear. This hole is visible for *R* > 0.8, hence the bar in the diagram from *R* = 0.8 to *R* = 1.0. (**c**) If we increase *R*, no perceptible changes are observed. Only one hole persists and it is reflected in the bar code below. (**d**) The hole disappears by formation of big triangles at about *R* = 2.8. From the observation of the bar code, we notice that the data set has the topology of a circle, with one single interior hole. In general, those holes or voids that persist the most reflect the persistent topology of the data.

In essence, simplicial homology reflects the number of holes in a given dimension for the simplicial complex. For instance, a chain of edges may close a hole, while the interior space within a tetrahedron is a void. If we record the precise value of *R* for which a hole or void (in any dimension) appears, and the *R* value for which it disappears—by the appearance of a filled triangle or tetrahedron, for instance—the resulting plots will indicate which topological features persist more. Those indicate the true topology of the data. For a graphical interpretation of this explanation, see Figure 2.

Under the TDA framework, noisy data produce topological structures with small persistence. The true topology (manifold structure) is described by these structures that persist the most. Once unveiled, the topological structure of data or, equivalently, the right shape of the data manifold, will allow us to interpolate data in the right topological space—in the tangent plane to the manifold at each datum—and not in Euclidean space.

### 2.3. Pseudo-Experimental Data—Learning a Visco-Hyperelastic Response

In this first example, we consider pseudo-experimental (numerical) data coming from a finite element simulation. We consider the same visco-hyperelastic material previously considered in [32], but this time altered with noise. This noise has a standard deviation of 10% of the mean value.

The considered material is assumed to obey a Mooney–Rivlin constitutive law

(7) $$W=C_{1}\left({\bar{I}}_{1}-3\right)+C_{2}\left({\bar{I}}_{2}-3\right)+D_{1}{\left(J-1\right)}^{2}$$

with ${\bar{I}}_{1}=J^{-\frac{2}{3}}I_{1}$ and ${\bar{I}}_{2}=J^{-\frac{4}{3}}I_{2}$. $I_{1}=\lambda_{1}^{2}+\lambda_{2}^{2}+\lambda_{3}^{2}$ and $I_{2}=\lambda_{1}^{2}\lambda_{2}^{2}+\lambda_{2}^{2}\lambda_{3}^{2}+\lambda_{3}^{2}\lambda_{1}^{2}$ are the invariants of the right Cauchy-Green tensor C. In turn, *J* represents the determinant of the gradient of deformation tensor. We took $C_{1}=27.56$ MPa, $C_{2}=6.89$ MPa, and $D_{1}=0.0029$ MPa.

The viscous part of the behavior is modeled after a Prony series expansion of the type

$$\begin{array}{cc} \frac{G(t)}{G_{0}} & =1-\sum_{i=1}^{2}{\bar{g}}_{i}^{P}\left(1-\exp\left(-\frac{t}{\tau_{i}}\right)\right)\\ \frac{K(t)}{K_{0}} & =1-\sum_{i=1}^{2}{\bar{k}}_{i}^{P}\left(1-\exp\left(-\frac{t}{\tau_{i}}\right)\right) \end{array}$$

with ${\bar{g}}_{i}^{P}=\left[0.2,0.1\right]$ and ${\bar{k}}_{i}^{P}=\left[0.5,0.2\right]$. The relaxation times take the values $\tau_{i}=\left[0.1,0.2\right]$ seconds, respectively. Initial instantaneous Young’s modulus and Poisson’s ratio corresponding to these values are E=206.7 MPa and ν=0.45, respectively.

With the material as just described, a plane-stress, biaxial experiment is reproduced. This experiment consists of two different loading steps, each one followed by a relaxation step. A total of 50 different data sets have been obtained for this experiment.

**A mean GENERIC model**. A regression procedure is then accomplished for each one of the 50 different experiments, so as to determine their precise GENERIC expression. With the obtained values, we first compute the mean GENERIC model by simply taking mean values for each one of the GENERIC model components. This “mean” GENERIC model is compared to the noise-free numerical experiment, taken as ground truth.

**Extracting the topology of data: GENERIC-TDA model**. Instead of just computing the mean values of each term of the GENERIC model, it seems judicious to employ TDA to unveil the topology of data and to determine the final GENERIC model by interpolating from the right neighboring experimental results. To this end, we considered a data set composed by 20 different loading states (all consisting of a load-relaxation-load-relaxation sequence) and the addition of noise (10% sdv) to each one of these processes, so as to obtain 50 different tests for each one of the 20 loading processes. This makes a total of one thousand different tests.

One of these tests (noise-free) is kept as the reference solution. We employ TDA to find the “neighboring” test to this reference solution and, by employing Kriging, to obtain the final numerical values of the GENERIC model. We would like to emphasize that interpolation is made not in the Euclidean space, but in the right tangent space to the data manifold, as unveiled by TDA techniques. Note that we speak of a tangent plane to a noisy manifold, since TDA is able to unveil the topology that persists the most, thus giving an apparent noise-free topology.

Weights for the interpolation are obtained by different Kriging interpolation techniques: Simple, Ordinary, and Local.

### 2.4. Learning the Constitutive Model of Porcine Carotid Tissue

One of the most intricate experimental procedures in the framework of solid mechanics is perhaps that of constitutive modeling of soft living tissues. Here, we will employ data previously obtained and presented in [14] for the constitutive modeling of porcine carotid tissue.

What is remarkable in soft living tissue modeling is, on one hand, the heterogeneity and anisotropy of the tissue; and on the other, the large differences between experimental values found in different specimens. For instance, the behavior of porcine carotid tissue—whose interest is to serve as a proxy of that of humans—differs strongly if the sample is extracted from proximal (i.e., close to the heart) positions of the vessel or if it is extracted from distal positions. Additionally, there is a strong anisotropy regarding circumferential versus longitudinal behavior.

In [14], a traditional fitting procedure was accomplished so as to determine the best fitting model for these data. Taking the mean of the experimental results arising from [14] as the only plausible reference solution—this is standard experimental procedure in the literature—what we did first was to determine by TDA the set of experimental results neighboring this (mean) reference solution. A GENERIC model was then determined for each one of these neighbor results. Three different approaches were then compared: (i) a GENERIC model whose terms are computed as the mean values of each of the GENERIC models for each neighboring curve (the closest ones in the data manifold); and either (ii) ordinary or (iii) local Kriging interpolation techniques among these neighbors of the terms of a new GENERIC model. The result of our approach is a new GENERIC-TDA model whose integration in (pseudo-)time produces a prediction of the tissue behavior.

**Experimental tests**. To introduce to the reader the most significant details in the experimental models used for our numerical analysis, here we show a brief description of the sample’s harvesting and tensile test protocols performed in [14]. The interested reader is referred to this article for a precise description of the experimental campaign.

We consider nine female pigs of 3.5±0.45 months (mean ± SD). The experiments on these swine were approved by the Ethical Committee for Animal Research of the University of Zaragoza. All procedures were carried out in accordance with the Principles of Laboratory Animal Care (86/609/EEC Norm, incorporated into Spanish legislation through the RD 1021/2005).

For each one of the left and right carotids, proximal and distal regions were considered for mechanical testing, as mentioned before. At each location, circumferential and longitudinal strips, approximately 3-mm-wide and 11-mm-long, and 5-mm wide and 15-mm long, respectively, were cut. A total of 14 carotid specimens with 47 and 49 valid tests were performed for the proximal and distal zones, respectively. A minimum number of two test strips along each direction was accomplished for each specimen.

**Figure 3:**
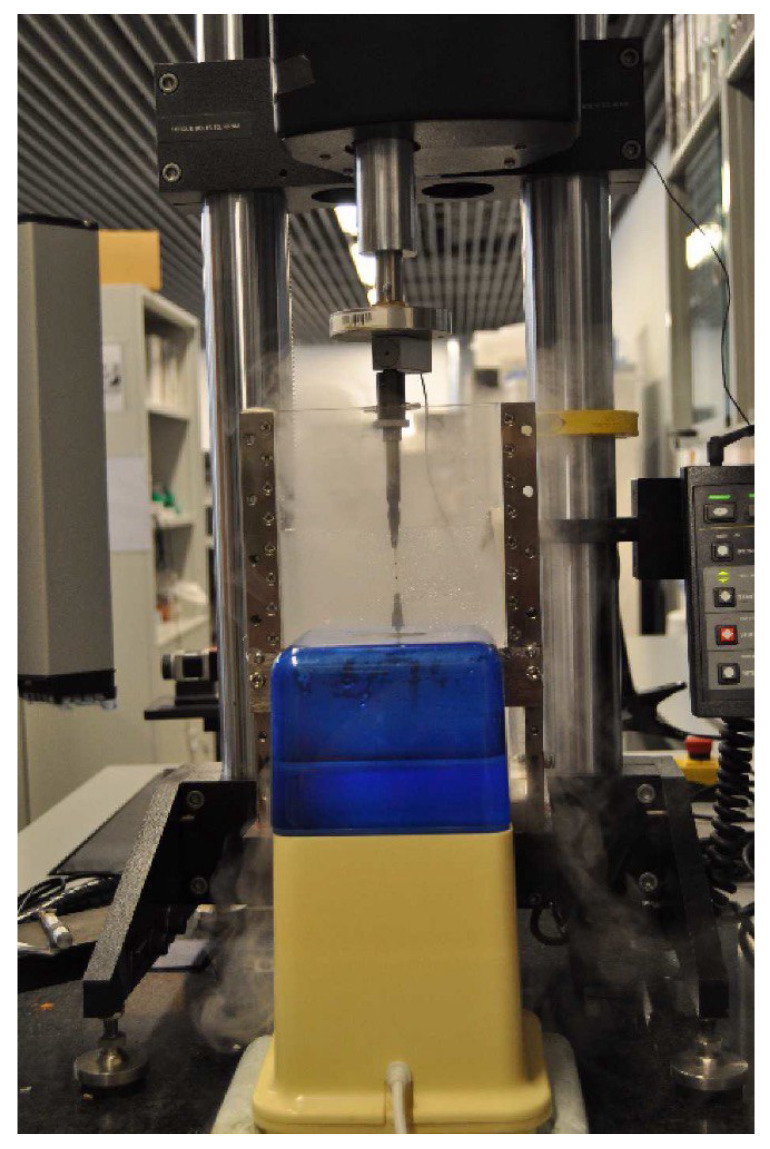
Experimental setup. Instron Microtester 5548 System with two clamps holding the sample. The samples are subjected to the tensile test under a humidity-controlled environment to prevent sample drying.

Simple tension tests of the carotid strips were performed in a high-precision-drive Instron Microtester 5548 system, see Figure 3. The procedure is properly described in [14], and consisted of the Instron Microtester 5548 System with two clamps holding the sample. The samples are subjected to the tensile test under a humidity-controlled environment to prevent sample drying.

The applied force was measured with a 5-N load cell with a minimal resolution of 0.001 N, and the axial strain was measured using a noncontact Instron 2663-281 video-extensometer equipped with a high-performance digital camera with a megapixel sensor (0.5 m ± 0.5% ).

Different loading and unloading cycles were applied that correspond to 60, 120, and 240 kPa (50%, 100%, and 200% of the estimated physiological stress state in the artery) at 30%/min of strain rate, which can be considered as quasi-static. Therefore, these experiments serve for the hyperelastic modeling of soft tissues, but not for their viscous characterization. The resulting GENERIC model will therefore be purely Hamiltonian under these conditions.

**Figure 4:**
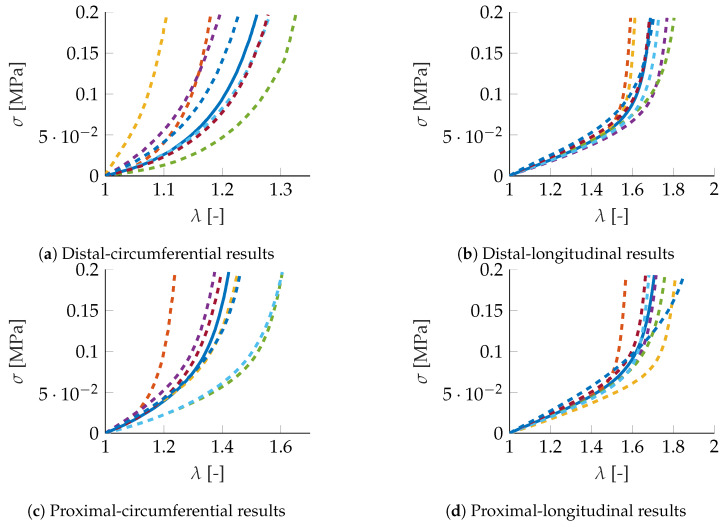
Experimental (**a**) distal-circumferential, (**b**) distal-longitudinal, (**c**) proximal-circumferential, and (**d**) proximal-longitudinal stress–stretch curves. The continuous blue line represents the mean values of the 14 experimental results, while the dashed lines represent the neighboring experiments, as found by TDA techniques.

For each one of the fourteen carotids, proximal and distal measurements are obtained, thus giving a total of 28 datasets. Each dataset includes circumferential and longitudinal stresses and stretches, $z=\{\sigma_{c},\lambda_{c},\sigma_{\ell },\lambda_{\ell }\}$. Due to the quasi-static nature of the experiments, time is actually a pseudo-time. In addition to these 14 stress–stretch curves for each location, a fifteenth curve is obtained by computing the mean value of the first 14. This curve will be taken as a sort of reference for comparison purposes, see Figure 4.

## 3. Results

### 3.1. Numerical Fitting of the Pseudo-Experimental Data Set

**Figure 5:**
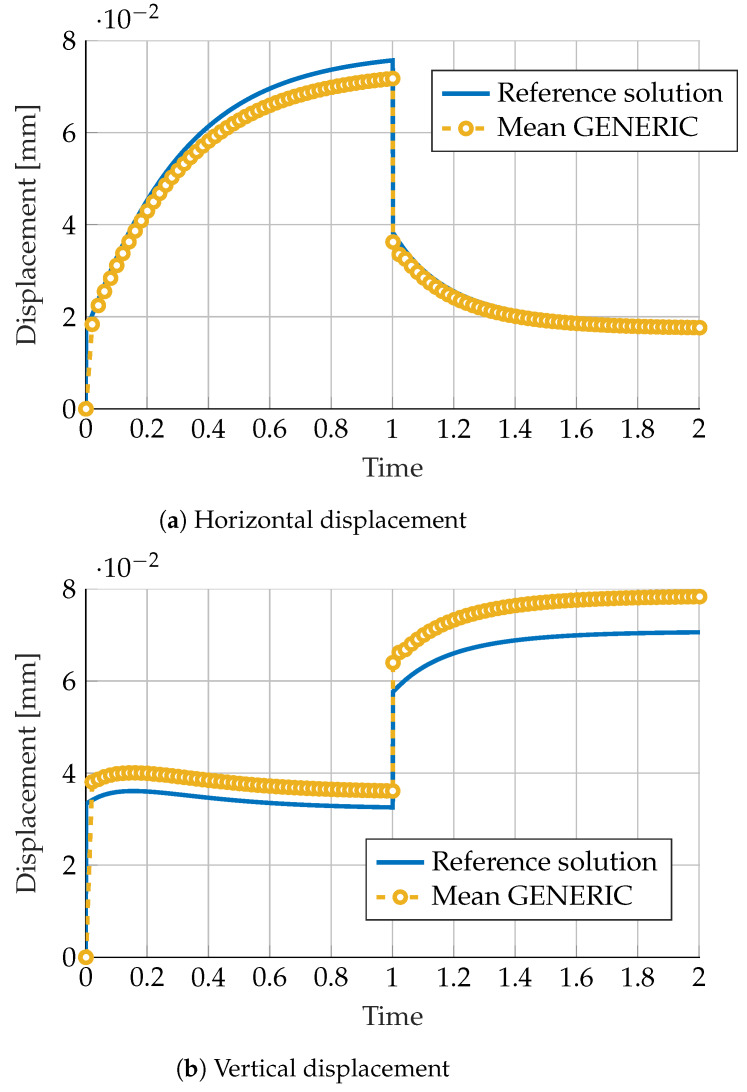
Comparison of (**a**) horizontal and (**b**) vertical displacement predicted by a General Equation for the Nonequilibrium Reversible-Irreversible Coupling (GENERIC) model obtained as the mean of 50 different noisy GENERIC models. Comparison with the noise-free reference solution in continuous blue line.

Recalling Section 2.3, Figure 5 shows the “mean” GENERIC model when compared to the noise-free numerical experiment.

As can be noticed from this figure, results show a poor accordance to the noise-free version of the data. Constructing a model by just computing the mean of each GENERIC model for noisy data seems not to be a good idea. If we consider it here, it is just because in the experimental framework, phenomenological models are very often obtained after computing means of the available results [14].

**Figure 6:**
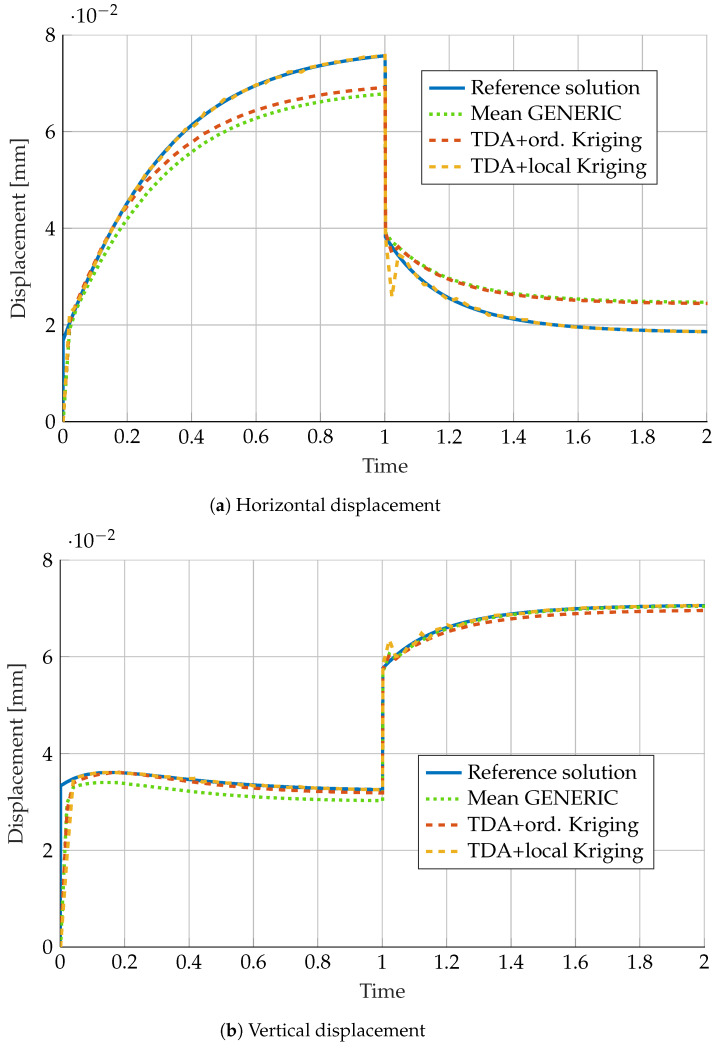
Comparison of (**a**) horizontal and (**b**) vertical displacement predicted by a GENERIC model obtained as the mean of 50 different noisy GENERIC models. Comparison with the noise-free reference solution in continuous blue line and the solutions obtained by Kriging interpolation between neighbors predicted by Topological Data Analysis.

Figure 6 shows a displacement comparison among the noise-free sample and the full GENERIC-TDA model with different Kriging interpolation techniques. Additionally, Table 1 shows the obtained 2-norm errors of the mentioned model results.

It is worth noting the high degree of accuracy obtained by employing local Kriging procedures. In combination with TDA, this procedure is able not just to filter the artificial noise added to the data, but to provide a very accurate GENERIC model able to reproduce the visco-hyperelastic model from which pseudo-experimental data was obtained.

### 3.2. Numerical Fitting of Porcine Carotid Tissue

**Figure 7:**
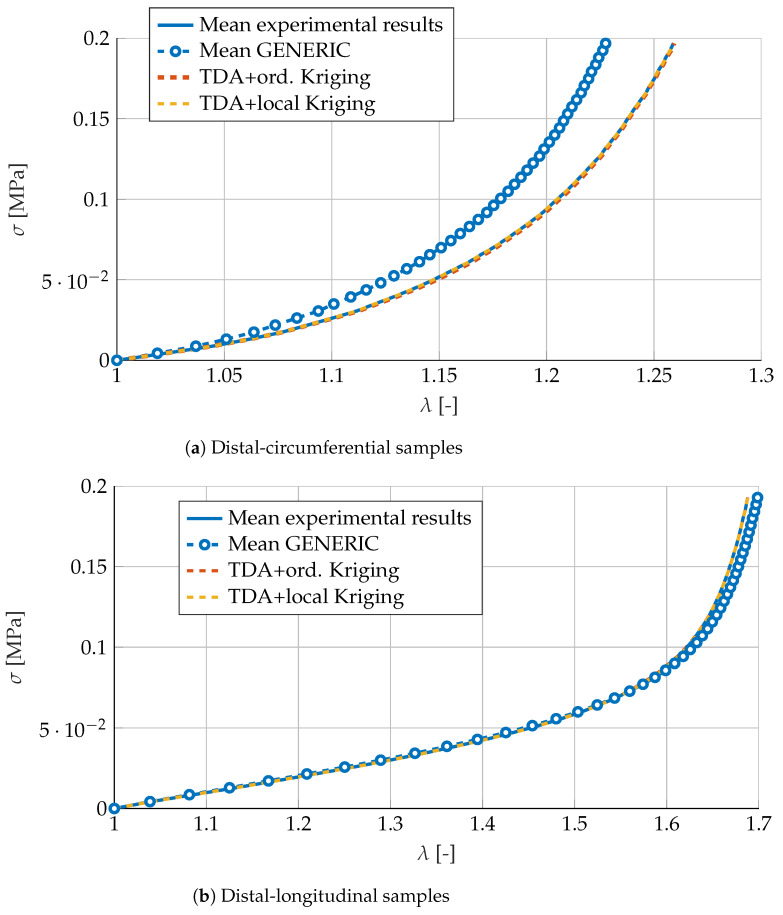
Comparison of (**a**) distal-circumferential and (**b**) distal-longitudinal models predicted by mean GENERIC values, or by Kriging interpolation of those samples neighboring the reference solution.

For the mean experimental curves (circumferential and longitudinal) of the distal samples, results are shown in Figure 7. It is worth noting that, in general, it seems not to be a good idea to just compute the mean values of the GENERIC models for each of the neighboring experimental curves. Particularly in the circumferential direction, the deviation from the reference solution is noteworthy. As in the previous section, results provided by Kriging interpolation outperform this approach. Particularly, local Kriging is found to provide the highest accuracy. The predicted behavior is almost indistinguishable from the mean experimental results.

**Figure 8:**
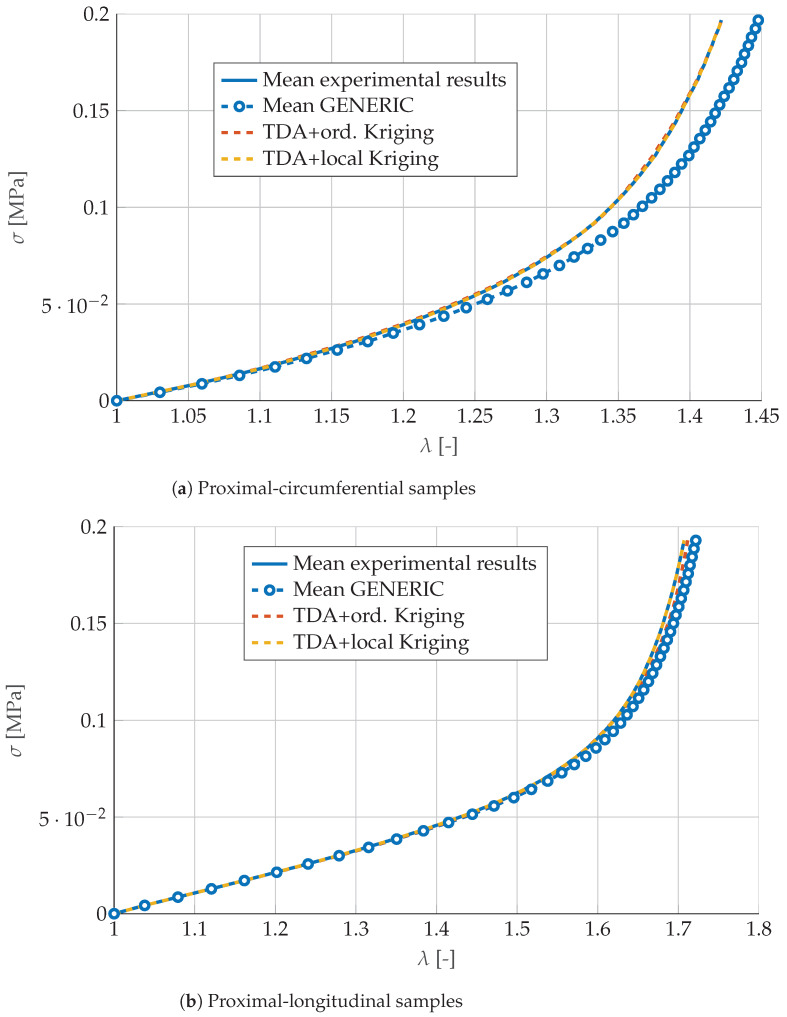
Comparison of (**a**) proximal-circumferential and (**b**) proximal-longitudinal models predicted by mean GENERIC values, or by Kriging interpolation of those samples neighboring the reference solution.

With the weights just computed for the distal samples, we constructed a new GENERIC model for the proximal results. Its predictions are shown in Figure 8. Once again, by just computing the mean of the GENERIC terms for the neighboring curves does not seem to produce good results. However, with the Kriging weights computed for the distal samples, results for the proximal samples are equally good. This demonstrates the robustness of the proposed approach.

It is worth noting that the computational cost of this procedure is by no means high: each sample took on average 2.52 seconds to obtain the corresponding GENERIC model.

## 4. Conclusions

In this paper, a new methodology for the data-driven learning of constitutive models is proposed. We made an emphasis on those cases in which large experimental deviations are present in the data. By first employing Topological Data Analysis techniques, we unveil the shape of the data manifold so as to allow us to perform interpolation on the right tangent plane to the manifold. Once the neighboring data are found, a GENERIC expression is found for the material under consideration. In other words, the precise form of the strain energy density and entropy potentials are found. This allows us to predict new loading states to a high degree of accuracy without the need to perform complex parameter fitting procedures to arrive to phenomenological models.

In addition, and in sharp contrast to other existing alternatives, our method is able to guarantee exact (to numerical precision) satisfaction of thermodynamic principles—conservation of energy and positive production of entropy—thanks to the GENERIC formalism.

To the best of our knowledge, no work has been performed in this line applied to biomedical living tissues. Despite the limitation of needing an admissible database to perform the learning process of our method, we strongly believe that the proposed GENERIC-TDA technique can be applied to the numerical fitting of highly nonlinear materials with sound accuracy, as shown in this manuscript. As a proof of concept, our results (developed in both synthetic and real experiments) show the high benefits of using data-driven models for materials simulation in fields where complex physical responses are present. We believe that machine learning methods combined with numerical modeling for biological systems (at any scale) is a very exciting young field with countless challenges and potential usefulness to both biomedical and numerical communities.

## Figures and Tables

**Table 1:** 2-norm errors in the obtention of the GENERIC model.

Mean GENERIC	2.24%
Simple Kriging	16.46%
Ordinary Kriging	1.82%
Local Kriging	0.38%
